# Tyrosine Phosphorylation of Tau by the Src Family Kinases Lck and Fyn

**DOI:** 10.1186/1750-1326-6-12

**Published:** 2011-01-26

**Authors:** Timothy ME Scales, Pascal Derkinderen, Kit-Yi Leung, Helen L Byers, Malcolm A Ward, Caroline Price, Ian N Bird, Timothy Perera, Stuart Kellie, Ritchie Williamson, Brian H Anderton, C Hugh Reynolds

**Affiliations:** 1 MRC Centre for Neurodegeneration Research, Department of Neuroscience, Institute of Psychiatry, King's College London, De Crespigny Park, Denmark Hill, London, SE5 8AF, UK; 2 Proteome Sciences plc, Institute of Psychiatry, King's College London, De Crespigny Park, Denmark Hill, London, SE5 8AF, UK; 3 Department of Neurology, CHU de Nantes, F-44000, France; 4 Yamanouchi Research Institute, Armstrong Road, Littlemore Park, Oxford, OX4 4SX, UK; 5 The University of Queensland, School of Chemistry and Molecular Biosciences, QLD4072, Australia; 6 Randall Division of Cell and Molecular Biophysics, King's College London, New Hunt's House, Guy's Campus, London. SE1 1UL, UK; 7 Institute of Child Health, University College London, Guilford Street, London WC1N 1EH, UK; 8 Division of Basic Medical Sciences, St. George's, University of London, Cranmer Terrace, London. SW17 0RE, UK; 9 Current Medicine Group, 11-21 Paul Street, London, EC2A 4JU, UK; 10 Johnson and Johnson Pharmaceutical Research and Development, Turnhoutsweg 30, B-2340 Beerse, Belgium; 11 Biomedical Research Institute, University of Dundee, Ninewells Medical School, Dundee, DD1 9SY, UK

## Abstract

**Background:**

Tau protein is the principal component of the neurofibrillary tangles found in Alzheimer's disease, where it is hyperphosphorylated on serine and threonine residues, and recently phosphotyrosine has been demonstrated. The Src-family kinase Fyn has been linked circumstantially to the pathology of Alzheimer's disease, and shown to phosphorylate Tyr18. Recently another Src-family kinase, Lck, has been identified as a genetic risk factor for this disease.

**Results:**

In this study we show that Lck is a tau kinase. *In vitro*, comparison of Lck and Fyn showed that while both kinases phosphorylated Tyr18 preferentially, Lck phosphorylated other tyrosines somewhat better than Fyn. In co-transfected COS-7 cells, mutating any one of the five tyrosines in tau to phenylalanine reduced the apparent level of tau tyrosine phosphorylation to 25-40% of that given by wild-type tau. Consistent with this, tau mutants with only one remaining tyrosine gave poor phosphorylation; however, Tyr18 was phosphorylated better than the others.

**Conclusions:**

Fyn and Lck have subtle differences in their properties as tau kinases, and the phosphorylation of tau is one mechanism by which the genetic risk associated with Lck might be expressed pathogenically.

## Background

The microtubule-associated protein tau is the main component of paired helical filaments (PHF) which are aggregated structures found in neurofibrillary tangles (NFT) in the brains of patients with Alzheimer's disease (AD). Neurofibrillary tangles are found in a number of other diseases termed 'tauopathies' which include frontotemporal dementia with Parkinsonism linked to chromosome 17 (FTDP-17), Pick's disease, progressive supranuclear palsy and corticobasal degeneration. The presence of tau deposits in all of these neurodegenerative diseases and particularly in FTDP-17, whose patients have mutations in the tau gene itself [1], suggests that tau protein may have an important role in the neurodegenerative process.

The tau in PHF (PHF-tau) is phosphorylated on over 40 serine and threonine residues [2-5] and this hyperphosphorylation hinders the ability of tau to bind to microtubules [6] and to promote microtubule assembly [7]. Several candidate kinases have been identified that can phosphorylate tau *in vitro*, including glycogen synthase kinase 3β (GSK-3β) [8,9]. GSK-3β can phosphorylate tau in cells, and inhibition of GSK-3β using lithium leads to a reduction in the degree of tau phosphorylation in neurons [10-13], which implies that GSK-3β is a physiological tau kinase.

**Figure 1:**
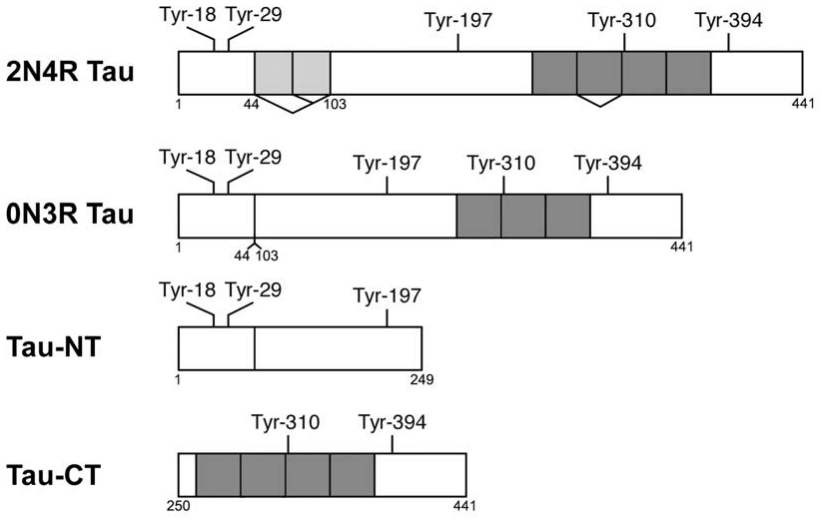
**Isoforms of tau and tau constructs used**. The shortest isoform (0N3R) lacks two N-terminal exons (shown in pale grey in 2N4R) and the second of the microtubule binding repeats (dark grey) due to alternative splicing. Tau-NT and Tau-CT represent the N-terminal and C-terminal halves of 0N4R tau. The numbering of residues in all constructs used is that of the 2N4R isoform of human tau.

The hyperphosphorylation of serines and threonines in PHF-tau is well documented, as is the lower level of phosphorylation in tau extracted from healthy adult or foetal brain [2,14,15]. However there is now evidence that phosphorylation of tyrosine residues in tau also occurs. We have shown that PHF-tau preparations from some AD cases contain tyrosine-phosphorylated tau [16,17], and Aβ peptide treatment of cultured neurons induced tyrosine phosphorylation of several proteins including tau [16]. Of the five tyrosine residues in human tau (Figure 1), phosphorylation of Tyr197 [18] and of Tyr394 [17] have been identified in PHF-tau, and of Tyr394 in foetal tau [17] using mass spectrometry. Tyrosine 18 was shown, using phosphospecific antibodies, to be phosphorylated in foetal and degenerating brain and in PHF, but not in healthy adult brain [19]. In tau-transfected fibroblasts and in SH-SY5Y human neuroblastoma cells the generic tyrosine phosphatase inhibitor, vanadate induced the phosphorylation of transfected tau primarily on Tyr394 [17]. Co-transfection of cells with tau kinases showed that Fyn and Syk phosphorylated tau principally on Tyr18, but Abl replicated the endogenous kinase activity by phosphorylating tau principally on Tyr394 [17,20]. Furthermore, in the JNPL3 mouse model of tauopathies which carries the tau P301L mutation, tyrosine phosphorylation at Tyr197 and Tyr394 was identified which increased with age and correlated with the formation of tau aggregates [18].

The Src family kinases, of which Fyn and Lck are members, are tyrosine kinases related to Src which was first identified as a viral protein (v-Src) in Rous sarcoma virus [21,22]. Src family kinases are characterised by six functional domains: the Src homology (SH) 4 domain; a unique region to each kinase; the SH3 domain; the SH2 domain; the catalytic domain and a negative regulatory C-terminal tail [23]. The family members Src, Fyn, Lck, Lyn and Yes are expressed in the CNS [24-29]. Initial evidence for the role of Src family kinases in AD was the discovery that a subset of neurons in AD brain show intense Fyn labelling compared to neurons in healthy brain [25]. Increased Fyn immunoreactivity has been shown in transgenic AD model mice [30]. Conversely, cognitive impairments were found in transgenic AD model mice overexpressing Fyn [31], and an increased level of Fyn in AD brains was correlated with cognitive impairment [32]. Brain slices and primary cortical neuronal cultures from Fyn knockout mice are resistant to the neurotoxic effects of amyloid-β oligomers [33,34], further implicating Src-family kinases in AD pathogenesis. Fyn is able to activate GSK-3β, which raises the possibility of Fyn being involved in the mechanism which leads to the serine and threonine hyperphosphorylation seen in AD [35]. Significant evidence, therefore, suggests a role for Fyn in AD. The Src family kinase member Lck, which is also present in neurons [26,27], has been reported to be down-regulated in AD [36], and interestingly the *Lck *gene locus has been reported to contain a risk factor for AD [37] located in Intron 1. This suggests that Lck may also play a role in the development of AD in affected individuals and this may be protective. The functional importance of the expression of Lck in neurons has also been demonstrated in Lck deficient mice where lack of Lck in retinal neurons causes development abnormalities in retinal architecture [38].

To address the issue of the role of Lck in AD, we investigated the phosphorylation of tau by Lck. Whilst Fyn and Lck had overlapping *in vitro *activity profiles, two main differences were found: (i) two-dimensional phosphopeptide maps and mass spetrometry revealed that Lck was somewhat better than Fyn at phosphorylating tyrosines other than Tyr18; and (ii) in co-transfected fibroblasts, mutation of tyrosines to phenylalanines interfered with phosphorylation by Lck of the remaining tyrosines. The *in vivo *activity profile of Lck contrasted with our previously reported findings where only the mutation of Tyr18 gave a marked reduction of phosphorylation in co-transfection experiments with Fyn. Together these differences suggest that Lck may interact with tau in a way that is subtly different from Fyn, with the possibility of a different role in generating pathology in AD.

## Results

### Analysis of tau phosphopeptides after *in vitro *phosphorylation

Phosphorylation of recombinant 2N4R tau *in vitro *by recombinant Lck or Fyn gave a stoichiometry of approximately 0.3 phosphates per mol of tau. This level of phosphorylation was expected to be adequate for site identification by mass spectrometry, while not being so high as to force the phosphorylation of non-physiological sites.

Tau phosphorylated *in vitro *using either recombinant Lck or Fyn was isolated by gel electrophoresis, and after trypsin digestion, the peptides were analysed by MALDI-ToF MS and LC-MS/MS. Phosphorylated peptides were identified by an increase of 80 Da in the mass of the peptide, and the location of the phosphate was confirmed in the second MS phase from sequence information given by collision-induced fragmentation. Additional File 1: Table S1 shows the peptides identified as containing phosphotyrosine residues in tau phosphorylated by Lck or Fyn. For a typical phosphopeptide analysis see additional File 2: Figure S1 and additional File 3: Table S2. For both Lck-phosphorylated and Fyn-phosphorylated tau, phosphopeptides containing each of the five tyrosines were found (tyrosines 18, 29, 197, 310 and 394). The positions of the phosphates were positively identified from the fragmentation patterns in the majority of cases, but in several cases ions in the MS survey scan did not meet the switching criteria to obtain MS/MS fragmentation data. Visual inspection of the MS survey scan allowed identification of the phosphorylated peptide by either comparison of the m/z and retention time of the ion with that of the corresponding phosphopeptide for which sequence information had been assigned (e.g. Fyn, Tyr394), or, in the case of Tyr29 by extraction of the ion at the m/z of the unphosphorylated peptide from both Fyn-phosphorylated and Lck-phosphorlyated tau which had been sequenced by MS/MS. The ion at m/z corresponding to the phosphorylated peptide was then extracted and a putative identification given if the retention time and charge state was equivalent to its unphosphorylated counterpart. The ratio of phosphorylated peptides to their non-phosphorylated equivalents suggested that the amount of phosphorylation of each residue was generally somewhat greater with the Lck preparation used than with Fyn.

### Two-dimensional phosphopeptide mapping of tau

**Figure 2:**
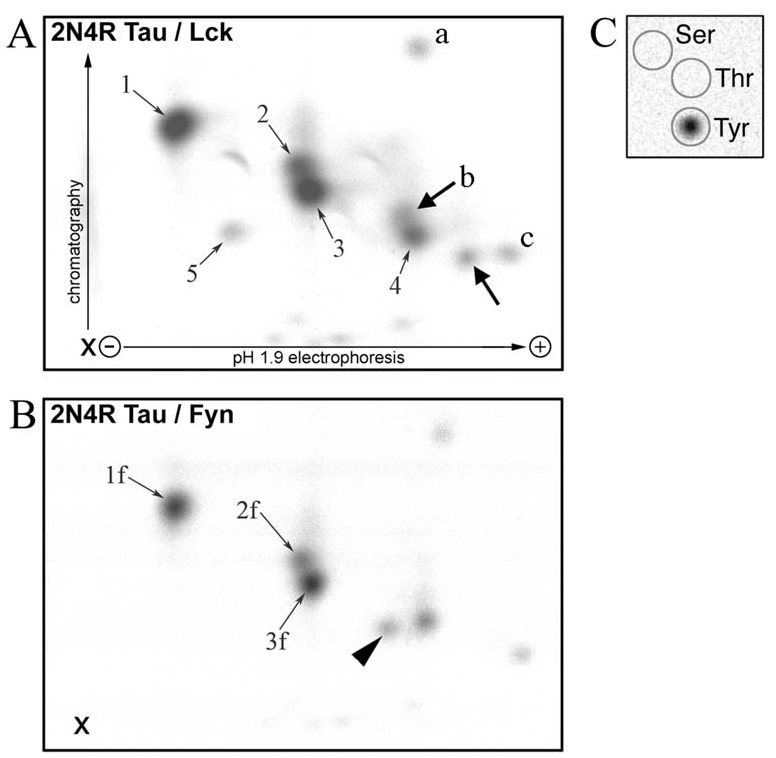
**2D Phosphopeptide mapping of tau phosphorylated by Lck or Fyn**. The origin of the samples is marked (X). *A*, Map of 2N4R tau phosphorylated by Lck. *B*, Map of 2N4R tau phosphorylated by Fyn. *Arrowhead *and *bold arrows *indicate phosphopeptide spots apparently unique to tau phosphorylated by Fyn and by Lck respectively. Numbered spots contained phosphopeptides which were identified by mass spectrometry (see additional File 4: Table S3). Spots 1, 2, 3, 4, 1f, 2f, and 3f were identified by matching the peptide masses measured by MALDI-ToF MS with the theoretical masses of peptides. Also, peptides and phosphotyrosines in spots 1, 2, 3, 4, 5 and 3f were identified by direct sequencing by MS/MS. Peptides in spots a, b and c were not identified by mass spectrometry. *C*, Phosphoamino acid analysis: a representative phosphoamino acid analysis as performed on eight of the Lck-generated phosphopeptide spots, where the circles show the outlines of the ninhydrin-positive phosphoamino acid standards. Spots thus analysed included 1, 3, 4 and 5, and those labelled a, b and c in Figure 3A, and all were found to contain only phosphotyrosine; a further phosphotyrosine-containing spot ran above spot 1 and so is not seen in 2*A*.

The LC-MS/MS data from Lck-phosphorylated and Fyn-phosphorylated tau were clearly similar. However, the sequence identification of phosphopeptides was incomplete and, to overcome the intrinsically non-quantitative nature of this technique, 2D phosphopeptide mapping was also used. Maps of tau peptides from Lck and Fyn phosphorylation are shown in Figure 2A and 2B, respectively. Eight spots generated by Lck (see Figure 2A) were analysed for phosphoamino acid content (exemplified in Figure 2C) and all eight were found to contain only phosphotyrosine. Although the 2D maps generated by Lck and Fyn had similar overlapping patterns, there were two additional spots generated by Lck (Figure 2A, bold arrows), one of which (labelled b) was shown chemically to contain phosphotyrosine, and one additional minor spot generated by Fyn (Figure 2B arrowhead). Their possible identities are considered later in this section.

In order to identify the phosphopeptides in the 2D spots, the cellulose layer containing the spots was scraped off, extracted, and analysed by mass spectrometry. Those spots where a phosphopeptide was successfully identified are indicated by numbers in Figure 2A and Figure 2B, and the identifications are shown in additional File 4: Table S3. Remarkably, spots 1-4 were all found to contain pTyr18. The three spots identified in Fyn-phosphorylated tau (1f, 2f and 3f) all contained the same similarly-migrating peptides produced by Lck (1, 2 and 3). It should be noted that, although not identified, from its position the spot to the right of the arrowheaded spot in Figure 2B is suspected to be equivalent to spot 4 on the Lck generated map (Figure 2A) and therefore also to contain Tyr18. None of the apparently unique peptides produced by Lck or Fyn (arrows and arrowhead) could be identified by mass-spectrometry. Control maps (not shown) with kinase but without tau did not produce spots, so those spots are likely to have originated from tau rather than from proteins in the kinase preparations.

Spot 5 in Figure 2A (from Lck-phosphorylated tau) was shown by LC-MS/MS sequencing to contain pTyr197. Thus, although Tyr197 was inferred only from the MS survey scan in the peptide mixture (see additional File 1: Table S1), analysis of this peptide spot clearly showed that Lck does phosphorylate this tyrosine residue. Longer exposure of 2D maps from Fyn-phosphorylated tau also demonstrated a radiolabelled phosphopeptide in this position (data not shown).

To further confirm the identity of spots, tau fragments containing only the N-terminal half of 0N tau (Tau-NT) or the C-terminal half of 4R tau (Tau-CT) (see Figure 1) were phosphorylated by Lck and the phosphopeptides were mapped (see additional File 5: Figure S2, A and B). The three main spots found from Tau-CT (arrowed) can be attributed to Tyr310 and Tyr394, and a mixing experiment (see additional File 5: Figure S2, C and D) shows their positions more clearly in relation to the other (i.e. NT-derived) peptides.

0N3R tau (the smallest CNS tau isoform) also contains all five tyrosines, and its 2D phosphopeptide map was very similar to that of 2N4R tau (not shown). However, due to its proximity to a splice junction, the phosphopeptide containing Tyr310 would have a different N-terminal portion for the two isoforms. One relatively hydrophobic peptide (the uppermost arrowed spot in Figure S2, A, C and D and labelled "a" in Figure 2A) was found in a different position in maps from the two isoforms. This spot therefore is likely to contain Tyr310 and suggests that the other spots present on the Tau-CT map, which includes the two arrowed spots in Figure 2A that were unique to the Lck generated map, are likely to contain Tyr394.

**Figure 3:**
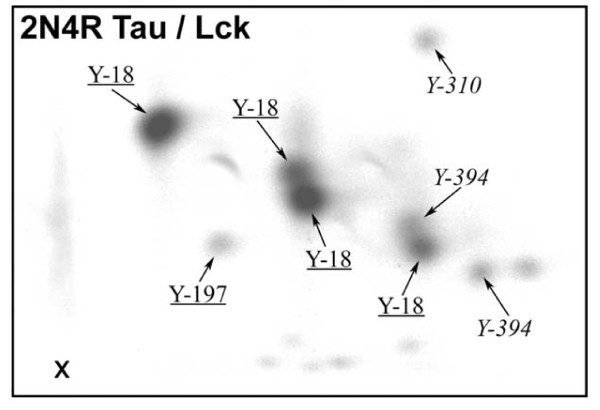
**Summary of phosphopeptide spot identification**. *Underlined *labels denote the phosphotyrosines in peptides identified in spots by a combination of MALDI-ToF MS and LC-MS/MS mass spectrometry. *Italics *show tentative identification of phosphotyrosines in spots by other methods. Unlabelled spots were not identified.

From a combination of these data the identity of many of the phosphopeptides can be deduced, shown in Figure 3., although one spot remained unidentified (labelled "c" in Figure 2A and left unlabelled in Figure 3). Although the results are self-consistent, spots may contain more than one peptide and so the possibility exists that the identifications by mass spectrometry may not be complete.

In the 2D phosphopeptide maps (Figures 2 and 3, and for peptide identity see additional File 4: Table S3) four prominent spots were each identified as containing pTyr18. This multiplicity was due to alternative trypsin cleavage sites around a consecutive pair of basic residues, and to the oxidation of methionine. Phosphorylation by Lck of the N-terminal fragment of tau (Tau-NT), which contains tyrosines 18, 29 and 197, incorporated much more radioactivity than phosphorylation of Tau-CT, even though no pTyr29 phosphorylation was detected and Tyr197 appears to be a minor site. This also suggests that Tyr18 is the major site phosphorylated by Lck. These results are consistent with the earlier results from transfection studies using Fyn [17].

Clearly, Lck and Fyn showed very similar phosphorylation patterns (Figure 2), with Tyr18 as the main site. The two unidentified peptides that appeared unique to Lck (Figure 2A, arrowed) migrated close to the peptides suggested to contain pTyr394. If they are indeed these pTyr394-containing peptides, this would suggest that Lck may phosphorylate Tyr394 rather better than Fyn does, but this requires confirmation. The greater radioactivity in Spot 5 with Lck (Figure 2A), compared to the equivalent one with Fyn (Figure 2B) which needed a longer exposure (described above), suggests that Tyr197 is a better site for Lck than for Fyn. The minor tau phosphopeptide that is apparently unique to Fyn was not identified (Figure 2B, arrowhead). Positions of spots on 2D maps provided confirmatory evidence for the identification of some of the phosphopeptides.

In summary; four of the five tyrosines in tau were positively identified as Lck phosphorylation sites, and two as Fyn sites (summarised in Table 1). The phosphopeptide containing the fifth tyrosine, Tyr29 (residues 24-44) was identified only from its mass and mass-difference on dephosphorylation; therefore the possibility that the phosphate was on Thr30 or Thr39 rather than on Tyr29 cannot be completely disregarded.

**Table 1:** Identification of phosphorylated tyrosines in tau by mass spectrometry after phosphorylation by Lck and Fyn

Phosphorylated Residue	Lck		Fyn	
	
	LC-MS/MS	^**a**^**2D-MS/MS**	LC-MS/MS	^**a**^**2D-MS/MS**
Tyr18	+	+	+	+
Tyr29	(ss)	-	(ss)	-
Tyr197	(ss)	+	(ss)	-
Tyr310	+	-	(ss)	-
Tyr394	+	-	+	-

+, site positively identified by MS/MS

(ss), survey scan (first MS spectrum) or MALDI-ToF MS; phosphopeptides identified by mass and mass-difference (phosphate loss) only.

^a ^LC-MS/MS analyses on phosphopeptides extracted from radiolabelled spots on 2D maps.

### Phosphorylation of tau by Lck in co-transfected cells

**Figure 4:**
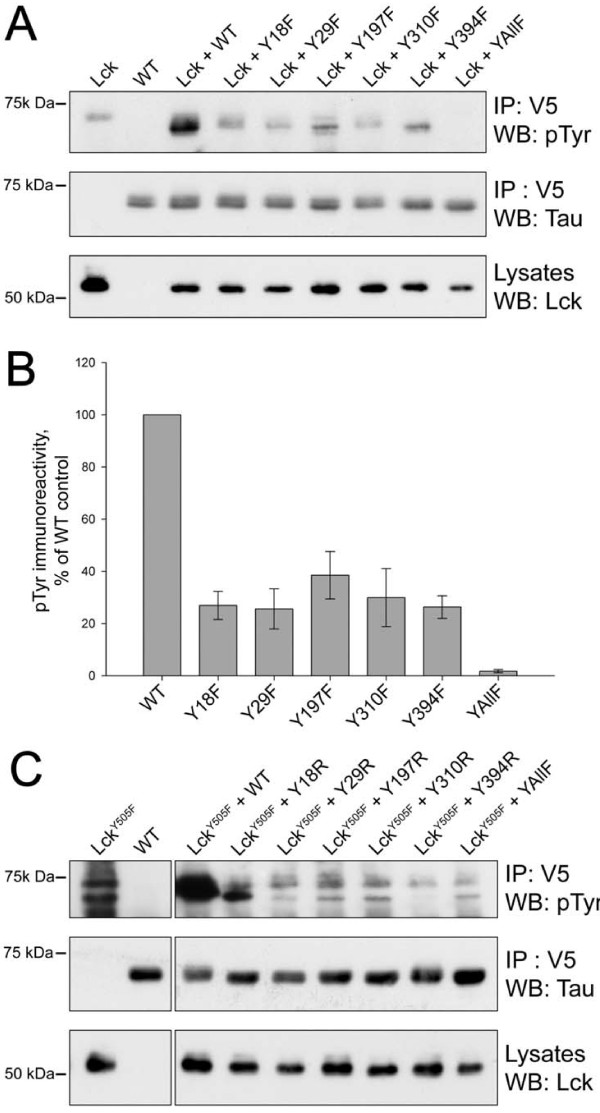
**Phosphorylation of tau by Lck in co-transfected COS-7 cells**. DNA constructs coding for V5-tagged tau 2N4R and for Lck were co-transfected into COS-7 cells. In A and B the tyrosine phosphorylation given by wild-type Lck of tau with single replacements of tyrosines is shown, while in C the phosphorylation by activated Lck (LckY505F) of tau with single tyrosines remaining is shown. Lysates were immunoprecipitated with anti-V5 antibody and Western blots were probed with 4G10 antiphosphotyrosine antibody (A top panel, and C) and with anti-tau antibody (A, middle panel), showing approximately equal immunoprecipitation of each tau construct; probing of lysates with anti-Lck antibody (A, bottom panel) showed that approximately equal amounts of Lck were expressed with each tau construct, although Lck alone gave stronger expression. B shows the amounts of immunoprecipitated tau phosphotyrosine after normalisation against immunoprecipitated tau (means of 3 experiments, +/- SEM). WT, wild-type tau (with all 5 tyrosines); YAllF, tau with all five tyrosines replaced by phenylalanines. In A and B, tau with single replacements of a tyrosine with phenylalanine are designated Y18F, Y29F, etc, and in C the mutants with a single tyrosine remaining are designated Y18R, Y29R, etc.

In order to determine if the phosphorylation of tau by Lck seen *in vitro *could also take place in cells, co-transfection experiments were undertaken. Western blotting with the phosphotyrosine specific antibody 4G10 revealed bands of tyrosine-phosphorylated proteins at approx. 68 kDa (Figure 4A, top panel), which co-migrated with the tau bands in the second panel. Multiple bands of tau are to be expected, due to mobility shifts resulting from phosphorylation on serines and threonines as well as tyrosine.

In order to identify which tyrosine or tyrosine residues were phosphorylated, tau constructs were used where each had one tyrosine converted to phenylalanine. A construct was also used, designated YAllF, where all five tyrosines had been converted to phenylalanine, which showed no detectable tyrosine-phosphorylated tau (Figure 4A). Replacement of any one tyrosine gave substantial reduction in tyrosine phosphorylation by Lck, but a basal level of phosphorylation was still detectable. This is in marked contrast to results with Fyn and Abl, where deletion of Y18 and Y394 ablated phosphorylation by Fyn and Abl respectively [17,20]. This suggests that multiple tyrosines can be phosphorylated simultaneously. Calculations from three experiments (Figure 4B) showed that mutation of any one tyrosine reduced the phosphorylation to 25-40% of that given by wild-type tau. This suggests therefore that the interaction of Lck with tau is disproportionately abrogated by removal of any one of the five tyrosine hydroxyl groups.

An alternative approach is to use tau containing only one tyrosine, the other four having been converted to phenylalanine [17], and compare these as substrates for phosphorylation. In view of the marked reduction seen by replacement of any one tyrosine (Figure 4, A and B), we anticipated that replacement of four would allow very little phosphorylation. Indeed, we could not detect any phosphorylation by wild-type Lck when it was co-transfected with any of the tau constructs with a single tyrosine remaining (results not shown). Therefore we repeated the experiment with an activated Lck mutant, LckY505F, where the inhibitory tyrosine phosphorylation site near the C-terminus, characteristic of Src-family kinases, had been mutated to phenylalanine. In tau that had only Tyr18 remaining there was a strongly phosphorylated band, but this was not as heavily phosphorylated as wild-type tau (Figure 4C). Results for the other tau mutants containing a single remaining tyrosine were more difficult to interpret, given that they closely resembled the banding pattern of phosphotyrosine for the TauYAllF mutant, in which no tyrosine residues are present. Looking at the phosphotyrosine banding patterns it is likely that there was a degree of background immunoprecipitation of the LckY505F, as the banding pattern seen in the phosphotyrosine immunoblot of the transfection with LckY505F alone resembles the pattern seen in the Tau Y29R, Y197R, Y310R and Y394R transfected samples. Although the results of this experiment are somewhat ambiguous, when taken together with the data from the transfections with single Tyr-Phe tau mutants (Figure 4A), these data suggest that Lck can phosphorylate multiple sites in tau within cells, and suggested that Tyr18 is a preferred site.

## Discussion

Tyrosine-phosphorylated tau has previously been identified in foetal brain and in PHF-tau from AD brain [16-18]. Phosphorylation of Tyr18 was identified using phosphospecific antibodies [19], while phosphorylation of Tyr197 and Tyr394 was identified using LC-MS/MS [17,18]. Our current studies are in agreement with previous reports that in co-transfected cells Fyn phosphorylates Tyr18 [17,19] while Abl phosphorylates Tyr394 [17]. Whilst a site-specific antibody [19] identified Tyr18 as a Fyn and Src phosphorylation site *in vitro*, such studies do not exclude the possibility of phosphorylation of other sites as well.

It has been reported that Lck mRNA is down regulated in AD brain, and more recently the Lck gene has been implicated as the locus for a possible genetic risk factor in AD; however, changes in protein expression or SNPs in Lck have not been fully characterised [36,37]. Although it might be expected that Lck phosphorylates tau in a manner similar to the other Src-family kinases Fyn and Src, the structural differences between these kinases might result in different substrate specificities. Therefore we undertook a study of tau phosphorylation by Lck to investigate this.

Phosphorylation of recombinant tau *in vitro *with Lck or Fyn shows that they are both able to phosphorylate several, possibly all, of the five tyrosines. However Tyr18, already known to be the favoured site for Fyn, Src and Syk, is also a site favoured by Lck. This is consistent with our results showing that when activated Lck (LckY505F) was co-transfected with tau constructs with only one tyrosine remaining, Tyr18 could be phosphorylated better than the other four tyrosines.

Using CHO cells co-transfected with Lck and tau, our results here indeed show that this kinase differs from Fyn [17] and Syk [20] in its ability to phosphorylate specific residues of tau. Wild-type human tau is phosphorylated in cells on tyrosine in a Lck-dependent manner, but this phosphorylation is considerably reduced by replacing any of the five tyrosines in human tau with phenylalanine. This is in contrast to earlier results with Fyn, Syk and Abl and it is unusual for replacement of tyrosine with phenylalanine to have a global effect on phosphorylation beyond what would be expected from removal of a phosphorylation site.

There are several possible reasons for the co-ordinated inhibition of phosphorylation at several different sites by mutation of one of them: 1. Conformational changes in tau structure caused by any one Tyr-to-Phe mutation; however this is unlikely as tau is a predominantly unstructured protein [39], and the tyrosines are distributed throughout the molecule. Nevertheless this is not entirely unprecedented: Src-family kinases have SH3 domains which can bind to tau [40-42], and Src-SH3 and Fyn-SH3 show some distinct differences in binding to various forms of tau that are hard to explain except by invoking conformational differences in tau [41]. 2. Lck may bind to phenylalanine (perhaps regarding that sequence as a pseudosubstrate) and so be inhibited. However, there is no evidence for this. 3. The Tyr-Phe mutations may alter phosphorylation on serines and threonines. There is little precedent for this: tyrosines 197 and 394 are in or near regions highly susceptible to multiple serine/threonine phosphorylation, but tyrosines 18, 29 and 310 are not. However, if altered Ser/Thr phosphorylation (e.g. at the sites, near Tyr197 and Tyr394 respectively) reduced the interaction of tau with Lck, then reduced phosphorylation at all five tyrosines might be expected.

The *in vitro *phosphorylation experiments had shown that Lck and Fyn phosphorylated tau in a similar manner. Lck appeared to be somewhat less selective than Fyn (or Syk) for Tyr18, but this would not account for the degree of difference seen in cells. The differences between the kinases seen in transfection experiments, therefore, may be due to differences in the behaviour of these kinases in cells. It is possible that other cellular proteins may bind to kinases or tau in a kinase-specific manner, affecting kinase action; there is a precedent for this, as 14-3-3 protein can bind to both GSK-3β and tau, affecting the latter's phosphorylation [43]. When considering differing substrate phosphorylation in cells it is also important to consider the cellular localisation of the kinases; for instance in neurons derived from embryonic stem cells Fyn and Src, have been show to be localised to cell bodies and neurites whereas Lck is localised solely to the cell body [44].

The sequence of tau is highly conserved in mammalian species in the C-terminal half of the molecule that contains the microtubule-binding regions, but shows more variation in the N-terminal half. Indeed, while humans and rhesus monkeys have two N-terminal tyrosines (18 and 29), other mammalian species (mouse, rat, cow and goat) have a 10 or 11 amino acid deletion in this region that includes one of the tyrosines [45], with the result that the remaining tyrosine is in a sequence context with features of both primate Tyr18 (on its N-terminal side) and primate Tyr29 (on its C-terminal side). Its ability to be phosphorylated in mouse brain as well as in human brain [19] suggests that Tyr18, rather than Tyr29, may be functionally the more important. Functions of the N-terminal part of tau, which has been termed the projection domain, have not been extensively studied, but it is reported to bind to membranes [46] and to other proteins including GSK-3β [47].

The serine/threonine phosphorylation sites on tau are predominantly in the central part of the molecule or near the C-terminus, on either side of the microtubule-binding repeats [3], and phosphorylation inhibits its binding to microtubules. Phosphorylation of Tyr18 does not directly affect the ability of tau to bind to microtubules [19], and may therefore have an alternative or additional role, e.g. in mediating or modulating the actions of the N-terminal projection domain. This role may be significant in brain development, as phosphorylation of Tyr18 was found in foetal but not in adult mouse brain [19]. The presence of phosphorylated Tyr18 [19], Tyr197 [18] and Tyr394 [17] in PHF-tau from AD brains but apparently not in normal adult brains suggests the possibility of a role in pathogenesis, paralleling that of serine/threonine hyperphosphorylation [48]. This is further indicated in the JNPL3 mouse model, where tau tyrosine phosphorylation increases with age of the animals and correlates with the development of tau aggregates [18].

It is not yet understood how the reported risk associated with the Lck gene locus or the down regulation of Lck mRNA impact on Lck activity. As the Src-family kinases appear to have broadly overlapping specificities for particular tyrosine residues on tau it is possible that localisation of the Src-family kinase is the determining factor for phosphorylation rather than their specificity for different tyrosine residues. It is possible that the loss of Lck-tau interactions and a concurrent increase in Fyn-tau interactions may result in tyrosine phosphorylation that may favour the development of AD pathology. This is in keeping with previous reports describing Fyn-tau interactions altering tau's cellular location [34,49] and could in some way explain the increased phosphorylation of Tyr197 and Tyr394 observed in AD brain. Much further work is needed to clarify these issues.

## Conclusions

By a combination approach of mass spectrometry and 2D phosphopeptide mapping four of the five tyrosines in tau (Tyr18, Tyr197, Tyr310 and Tyr394) were positively identified as *in vitro *Lck phosphorylation sites and the fifth (Tyr29) was identified as a probable Lck phosphorylation site. Two tyrosines (Tyr18 and Tyr197) were positively identified as *in vitro *Fyn phosphorylation sites and Tyr29, Tyr310, Tyr394 as probable phosphorylation sites. In co-transfection studies in cells, Lck showed no preferential for phosphorylation of any of the five tyrosines over one another, with all tyrosines appearing to be phosphorylated. This is in contrast to similar studies using Fyn, Syk or Abl and may be indicative of a differing role for Lck in AD pathogenesis.

## Methods

### Materials

Human 2N4R tau and 0N3R tau constructs were donated by M. Goedert (MRC Laboratory of Molecular Biology, Cambridge, UK). Tau N-terminal and C-terminal constructs were donated by H. Yanagawa (Mitsubishi Kasei Institute of Life Sciences, Tokyo, Japan). 2N4R tau, 0N3R tau and tau N- and C-terminal constructs were expressed in *Escherichia coli *and purified as previously described [50], with the exception that for 2N4R and 0N3R tau the final Mono S chromatography was omitted. The tau constructs used in this study are shown in Figure.1. For mammalian expression, V5His-tagged 2N4R tau in pcDNA3.1 was used, with Tyr-Phe mutants prepared by site-directed mutagenesis as described previously [17].

Lck and Fyn proteins were expressed in Sf9 cells using baculovirus constructs. The Lck construct had a C-terminal His-tag, while the Fyn construct (a gift from D. Markby, Sugen, San Francisco, CA, USA) was wild-type. Lck protein was purified using a cobalt containing matrix (Talon, Clontech, Palo Alto, CA, USA) and Fyn by an immunoaffinity column using Fyn3 antibody (Santa Cruz Biotechnology, Santa Cruz, CA, USA) with elution by its cognate peptide. Preliminary experiments were carried out using commercial preparations of Fyn and Src (Millipore, MA, USA). Lck constructs for mammalian expression (both wild-type and activated by the Y505F mutation) in the vector pCI were a gift from M. Bijlmakers (King's College London) [51].

Trypsin used was Sequencing Grade Modified Trypsin (Promega, Southampton, UK). Coomassie Blue staining of polyacrylamide gels was carried out using Brilliant Blue G (Sigma, Gillingham, UK). 2D Phosphopeptide mapping was carried out on 20 × 20 cm cellulose coated plates (Merck VWR, Poole, Dorset, UK). The anti-tau antibodies used were TP70 which has been described previously [52], and rabbit anti-human tau from DAKO (Ely, Cambridgeshire, UK). The monoclonal anti-phosphotyrosine antibody 4G10 was obtained from Upstate, and anti-V5 monoclonal antibody was obtained from Invitrogen (Paisley, UK). Monoclonal anti-Lck antibody 3A5 (sc-433) was obtained from Santa Cruz. Other reagents for cell culture and immunoprecipitation were as described previously [17].

### *In vitro *phosphorylation of tau

Phosphorylation was carried out in a volume of 20 μl, which included 5 μl of tau solution (containing 5 μg of tau), 5 μl of Lck or Fyn preparation and 10 μl of other components, to give a final concentration of 50 μM ATP, 3 μCi [γ^32^P]ATP if required, 20 mM MgCl_2_, 5 mM MnCl_2_, 0.1 mM EDTA, 1 mM DTT and 50 mM Tris-HCl pH 7.5. The phosphorylation mixture was incubated for 3 hours at 30°C. Following incubation the samples were placed in a boiling water bath for 5 min and then placed on ice for 10 min. After centrifuging at 16,000 × g for 5 min to remove precipitated protein, 10 μl SDS-PAGE sample buffer was added to the supernatants, which were boiled for a further 5 min and re-centrifuged before loading onto the gel.

### 2D Phosphopeptide mapping

Tau (2 μg) that had been tyrosine phosphorylated by Lck or Fyn was resolved by electrophoresis on 10% polyacrylamide gels and then transferred by semi-dry blotting onto Immobilon P (PVDF) membrane (Millipore, Watford, UK) at 15 V for 45 min. Protein was visualised initially by staining the membrane with 0.1% (wt/vol) amido black solution and the radiolabelled protein bands were located using a Fuji FUJIX BAS 1000 imaging system. The phosphorylated tau bands were cut out and counted using Cerenkov radiation. 2D Phosphopeptide mapping was carried out as described by Boyle et al [53]. Briefly, the excised bands were washed twice in 200 μM NaOH, then blocked in 0.5% (vol/vol) polyvinylpyrrolidine in 0.1 M acetic acid for 1 hour at 37°C, followed by five brief washes in H_2_O. Trypsin (1 μg) in 10 μl 0.2 M ammonium bicarbonate (pH 8.0) was added to the membrane strips. After trypsin treatment for 8 hours at 37°C a further 10 μl (1 μg) of trypsin solution was added and the strips were incubated for an additional 4 hours at 37°C. The surrounding liquid was then removed to a fresh tube and counted by Cerenkov radiation. Water (400 μl) was added to the samples which were then dried by vacuum centrifugation (Speed-Vac). The drying was repeated with 1 ml of H_2_O and then twice with 400 μl of pH 1.9 electrophoresis buffer (2.8% vol/vol formic acid, 7.8% vol/vol glacial acetic acid, 89.4% vol/vol H_2_O). The samples were dissolved in 10 μl of pH 1.9 electrophoresis buffer and loaded onto cellulose coated glass plates (20 cm × 20 cm). In the first dimension peptides were separated by electrophoresis at pH 1.9 at 1 kV for 1.25 hours. The second dimension consisted of chromatography in phospho-chromatography buffer (37.5% vol/vol *n*-butanol, 25% vol/vol pyridine, 7.5% vol/vol glacial acetic acid, 30% vol/vol H_2_O) for 18-20 hours. The maps were then visualised by imaging or autoradiography.

### Phosphoamino acid analysis

The required spots were scraped from the cellulose coated glass plates and 200 μl of 20% (vol/vol) acetonitrile was added to each sample of cellulose powder. The samples were mixed thoroughly, placed in a sonicating bath for 10 minutes, and centrifuged at 16,000 × g for 5 min. The supernatants were removed to fresh tubes and the process was repeated, and the second supernatants were pooled with the first. The samples were dried by vacuum centrifugation (Speed-Vac), dissolved in 100 μl of 6M HCl and hydrolysed for 1 hour at 110°C. The samples were again dried in the Speed-Vac, counted using Cerenkov radiation and dissolved in 5 μl of pH 1.9 electrophoresis buffer. 2 μl of phosphoamino acid standards (0.3 mg/ml each of phosphoserine, phosphothreonine and phosphotyrosine in water) was added. The samples were loaded onto cellulose coated glass plates (4 samples per plate) and separated in the first dimension by electrophoresis at pH 1.9 at 1 kV for 40 min and in the second dimension by electrophoresis at pH 3.5 (using pH 3.5 buffer which consisted of 5% (vol/vol) glacial acetic acid, 0.5% (vol/vol) pyridine, 94.5% (vol/vol) H_2_O) at 1 kV for 25 min [53]. The plates were sprayed with 0.25% (wt/vol) ninhydrin in acetone and placed in an oven at 160°C for 1 min to reveal the position of the amino acid standards. The positions of the radiolabelled amino acids were visualised by imaging and compared to the positions of the non-radioactive standards.

### Analysis of tau peptides by mass spectrometry

Samples of tyrosine phosphorylated tau (phosphorylated either by Lck or Fyn) as well as unphosphorylated tau were resolved by SDS-PAGE and stained with Coomassie Brilliant Blue G, and the required band was cut out of the gel.

For MALDI-ToF MS (matrix assisted laser desorption/ionisation time of flight mass spectrometry) analysis, cysteine residues were reduced with 10 mM DTT in 100 mM ammonium bicarbonate (56°C for 30 min) and derivatised by treatment with 55 mM iodoacetamide (room temperature for 20 min) to form stable carbamidomethyl derivatives. Trypsin digestion was carried out overnight at room temperature after an initial 1 hour incubation at 37°C. The digested samples were desalted and concentrated using ZipTipC18 microtips (Millipore, Bedford, MA, USA). Peptides were eluted in 4 μl 50% (vol/vol) acetonitrile/0.1% (wt/vol) trifluoroacetic acid, and 0.5 μl was loaded onto a target plate with 0.5 μl matrix (α-cyano-4-hydroxy-cinnamic acid). Peptide mass fingerprints were acquired in positive ion mode and reflectron mode with delayed extraction on a Voyager DE-Pro instrument (Applied Biosystems, Foster City, CA, USA). An autolytic tryptic peptide of mass 2163.0569 Da was used as an internal mass standard, resulting in a mass accuracy <50 ppm.

For LC-MS/MS (liquid chromatography tandem mass spectrometry) analysis, samples were prepared as described above for MALDI-ToF MS, and peptides were then extracted from SDS-PAGE gel pieces by incubating with 50 mM ammonium bicarbonate at 37° for 15 min followed by acetonitrile for 15 min, and repeating this cycle once. The extracts were pooled and dried in a Speed-Vac. Each sample was then resuspended in 23 μl of 50 mM ammonium bicarbonate and analysed. Chromatographic separations were performed using an Ultimate LC system (Dionex, Camberley, UK). Peptides were resolved by reverse phase chromatography on a 75 μm C18 PepMap column (Dionex). A gradient of acetonitrile in 0.05% (vol/vol) formic acid was delivered to elute the peptides at a flow rate of 200 nl/min. Peptides were ionised by electrospray ionisation using a Z-spray source fitted to a QToF-*micro *mass spectrometer (Waters Ltd, Elstree, UK). The instrument was set to run in automated switch mode, selecting precursor ions based on their intensity and charge, for sequencing by collision-induced fragmentation. The MS/MS analyses were conducted using collision energy profiles that were chosen based on the m/z and the charge state of the peptide; a total of nine individual MS/MS spectra were combined for each precursor.

The mass spectral data were processed into peptide mass lists (MALDI-ToF MS data) and peak lists (MS/MS data) and searched against the full length sequence of human tau using Mascot software (Matrix Science, London, UK). Tyrosine phosphorylated peptides were identified by selecting this as a variable modification within the searching parameters. Serine and threonine phosphorylation was also included in the search. The exact location of the modification within each peptide was determined by the pattern of fragment ions produced.

### Analysis of phosphopeptide spots from 2D maps by mass spectrometry

Major spots were identified by imaging of radioactivity and the area corresponding to the spot on the cellulose was scraped from the plate. Peptides were extracted from the cellulose using 50% (vol/vol) MeOH and 0.05% (vol/vol) formic acid in water, and then dried in a Speed-Vac. The samples were then analysed by MALDI-ToF and LC-MS/MS as detailed above.

### Transfection and harvesting of fibroblasts, immunoprecipitations and Western blotting

Culturing, transfection and harvesting of COS-7 cells was carried out as described previously [17]. Cells were harvested 24 h after transfection into SDS-PAGE sample buffer [54], and immunoprecipitations and Western analysis were carried out as described previously [17,55]. After visualisation of blots using enhanced chemiluminescence, films were scanned using an Epson Perfection 4990 scanner (Hemel Hempstead, UK) and quantified using Phoretix 1D Plus software (Nonlinear Dynamics, Newcastle upon Tyne, UK).

## Abbreviations used

Aβ: amyloid-beta; AD: Alzheimer's disease; APP: amyloid precursor protein; DTT: dithiothreitol; FTDP-17: frontotemporal dementia with Parkinsonism linked to chromosome 17; GSK-3: glycogen synthase kinase 3; LC: liquid chromatography; LC-MS/MS: liquid chromatography tandem mass spectrometry; MALDI-ToF MS: matrix assisted laser desorption/ionisation time-of-flight mass spectrometry; NFT: neurofibrillary tangles; PHF: paired helical filaments; PHF-tau: tau in paired helical filaments; PP2: 4-amino-5-(4-chlorophenyl)-7(*t*-butyl)pyrazol(3,4-D)pyramide; PVDF: polyvinylidene difluoride; SDS-PAGE: sodium dodecyl sulphate - polyacrylamide gel electrophoresis; Tau-CT: Tau C-terminal construct; Tau-NT: Tau N-terminal construct;

## Competing interests

The authors declare that they have no competing interests.

## Authors' contributions

TMES, PD, KL and HLB carried out experiments and analysed data; TMES, PD, KL, HB, MAW, CP, INB, TP, SK, RW, BHA and CHR designed experiments; CHR, RW and TMES wrote the manuscript. All authors read and approved the manuscript.

## Supplementary Material

Additional file 1 **Table S1**. "Tyrosine-phosphorylated peptides identified by LC-MS/MS in tryptic digests of 2N4R tau after phosphorylation by Lck and Fyn." Click here for file

Additional file 2 **Figure S1**. "LC-MS/MS spectrum of a phosphopeptide of Mr 2148.83 Da from Lck-phosphorylated tau." Click here for file

Additional file 3 **Table S2**. "Analysis of LC-MS/MS spectrum shown in additional File 2: Figure S1." Click here for file

Additional file 4 **Table S3**. "Identification by mass spectrometry of tau phosphopeptides in spots from 2D phosphopeptide maps." Click here for file

Additional file 5 **Figure S2**. "2D Phosphopeptide mapping of tau C- and N-terminal constructs phosphorylated by Lck." Click here for file

## References

[B1] Hutton M Lendon CL Rizzu P Baker M Froelich S Houlden H Pickering-Brown S Chakraverty S Isaacs A Grover A Association of missense and 5'-splice-site mutations in *tau *with the inherited dementia FTDP-17 Nature 1998 393 702 705 10.1038/31508 9641683

[B2] Morishima-Kawashima M Hasegawa M Takio K Suzuki M Yoshida H Watanabe A Titani K Ihara Y Hyperphosphorylation of tau in PHF Neurobiol Aging 1995 16 365 380 10.1016/0197-4580(95)00027-C 7566346

[B3] Hanger DP Betts JC Loviny TLF Blackstock WP Anderton BH New phosphorylation sites identified in hyperphosphorylated tau (paired helical filament-tau) from Alzheimer's disease brain using nanoelectrospray mass spectrometry J Neurochem 1998 71 2465 2476 10.1046/j.1471-4159.1998.71062465.x 9832145

[B4] Collins MO Yu L Coba MP Husi H Campuzano I Blackstock WP Choudhary JS Grant SG Proteomic analysis of in vivo phosphorylated synaptic proteins J Biol Chem 2005 280 5972 5982 10.1074/jbc.M411220200 15572359

[B5] Hanger DP Byers HL Wray S Leung KY Saxton MJ Seereeram A Reynolds CH Ward MA Anderton BH Novel phosphorylation sites in tau from Alzheimer brain support a role for casein kinase 1 in disease pathogenesis J Biol Chem 2007 282 23645 23654 10.1074/jbc.M703269200 17562708

[B6] Biernat J Gustke N Drewes G Mandelkow EM Mandelkow E Phosphorylation of Ser^262 ^strongly reduces binding of tau to microtubules: Distinction between PHF-like immunoreactivity and microtubule binding Neuron 1993 11 153 163 10.1016/0896-6273(93)90279-Z 8393323

[B7] Lindwall G Cole RD Phosphorylation affects the ability of tau protein to promote microtubule assembly J Biol Chem 1984 259 5301 5305 6425287

[B8] Lovestone S Reynolds CH The phosphorylation of tau: A critical stage in neurodevelopment and neurodegenerative processes Neuroscience 1997 78 309 324 10.1016/S0306-4522(96)00577-5 9145789

[B9] Buée L Bussière T Buée-Scherrer V Delacourte A Hof PR Tau protein isoforms, phosphorylation and role in neurodegenerative disorders Brain Res Rev 2000 33 95 130 10967355 10.1016/s0165-0173(00)00019-9

[B10] Hong M Chen DCR Klein PS Lee VMY Lithium reduces tau phosphorylation by inhibition of glycogen synthase kinase-3 J Biol Chem 1997 272 25326 25332 10.1074/jbc.272.40.25326 9312151

[B11] Muñoz-Montaño JR Moreno FJ Avila J Díaz-Nido J Lithium inhibits Alzheimer's disease-like tau protein phosphorylation in neurons FEBS Lett 1997 411 183 188 9271202 10.1016/s0014-5793(97)00688-1

[B12] Xie H Litersky JM Hartigan JA Jope RS Johnson GV The interrelationship between selective tau phosphorylation and microtubule association Brain Res 1998 798 173 183 10.1016/S0006-8993(98)00407-7 9666118

[B13] Lovestone S Davis DR Webster MT Kaech S Brion JP Matus A Anderton BH Lithium reduces tau phosphorylation: Effects in living cells and in neurons at therapeutic concentrations Biol Psychiatry 1999 45 995 1003 10.1016/S0006-3223(98)00183-8 10386182

[B14] Garver TD Harris KA Lehman RAW Lee VM-Y Trojanowski JQ Billingsley ML t phosphorylation in human, primate, and rat brain: Evidence that a pool of t is highly phosphorylated in vivo and is rapidly dephosphorylated in vitro J Neurochem 1994 63 2279 2287 10.1046/j.1471-4159.1994.63062279.x 7964748

[B15] Matsuo ES Shin RW Billingsley ML Van DeVoorde A O'Connor M Trojanowski JQ Lee VM-Y Biopsy-derived adult human brain tau is phosphorylated at many of the same sites as Alzheimer's disease paired helical filament tau Neuron 1994 13 989 1002 10.1016/0896-6273(94)90264-X 7946342

[B16] Williamson R Scales T Clark BR Gibb G Reynolds CH Kellie S Bird IN Varndell IM Sheppard PW Everall I Anderton BH Rapid tyrosine phosphorylation of neuronal proteins including tau and focal adhesion kinase in response to amyloid-beta peptide exposure: involvement of Src family protein kinases J Neurosci 2002 22 10 20 11756483 10.1523/JNEUROSCI.22-01-00010.2002 PMC6757621

[B17] Derkinderen P Scales TM Hanger DP Leung KY Byers HL Ward MA Lenz C Price C Bird IN Perera T Tyrosine 394 is phosphorylated in Alzheimer's paired helical filament tau and in fetal tau with c-Abl as the candidate tyrosine kinase J Neurosci 2005 25 6584 6593 10.1523/JNEUROSCI.1487-05.2005 16014719 PMC6725430

[B18] Vega IE Cui L Propst JA Hutton ML Lee G Yen SH Increase in tau tyrosine phosphorylation correlates with the formation of tau aggregates Brain Res Mol Brain Res 2005 138 135 144 10.1016/j.molbrainres.2005.04.015 15913839 PMC3677942

[B19] Lee G Thangavel R Sharma V Litersky J Bhaskar K Fang S Do L Andreadis A Van Hoesen G Ksiezak-Reding H Phosphorylation of tau by fyn: implications for Alzheimer's disease J Neurosci 2004 24 2304 2312 10.1523/JNEUROSCI.4162-03.2004 14999081 PMC6730442

[B20] Lebouvier T Scales TM Hanger DP Geahlen RL Lardeux B Reynolds CH Anderton BH Derkinderen P The microtubule-associated protein tau is phosphorylated by Syk Biochim Biophys Acta 2008 1783 188 192 10.1016/j.bbamcr.2007.11.005 18070606 PMC2258316

[B21] Brugge JS Erikson RL Identification of a transformation-specific antigen induced by an avian sarcoma virus Nature 1977 269 346 348 10.1038/269346a0 198667

[B22] Levinson AD Oppermann H Levintow L Varmus HE Bishop JM Evidence that the transforming gene of avian sarcoma virus encodes a protein kinase associated with a phosphoprotein Cell 1978 15 561 572 10.1016/0092-8674(78)90024-7 214242

[B23] Brown MT Cooper JA Regulation, substrates and functions of src Biochim Biophys Acta 1996 1287 121 149 8672527 10.1016/0304-419x(96)00003-0

[B24] Cotton PC Brugge JS Neural tissues express high levels of the cellular src gene product pp60c-src Mol Cell Biol 1983 3 1157 1162 6192323 10.1128/mcb.3.6.1157-1162.1983 PMC368645

[B25] Shirazi SK Wood JG The protein tyrosine kinase, fyn, in Alzheimer's disease pathology Neuroreport 1993 4 435 437 10.1097/00001756-199304000-00024 8388744

[B26] Omri B Crisanti P Marty MC Alliot F Fagard R Molina T Pessac B The Lck tyrosine kinase is expressed in brain neurons J Neurochem 1996 67 1360 1364 10.1046/j.1471-4159.1996.67041360.x 8858916

[B27] Van Tan H Allee G Benes C Barnier JV Vincent JD Fagard R Expression of a novel form of the p56lck protooncogene in rat cerebellar granular neurons J Neurochem 1996 67 2306 2315 10.1046/j.1471-4159.1996.67062306.x 8931462

[B28] Umemori H Wanaka A Kato H Takeuchi M Tohyama M Yamamoto T Specific expressions of Fyn and Lyn, lymphocyte antigen receptor-associated tyrosine kinases, in the central nervous system Brain Res Mol Brain Res 1992 16 303 310 10.1016/0169-328X(92)90239-8 1337939

[B29] Sudol M Hanafusa H Cellular proteins homologous to the viral yes gene product Mol Cell Biol 1986 6 2839 2846 3491292 10.1128/mcb.6.8.2839-2846.1986 PMC367851

[B30] Tomidokoro Y Ishiguro K Harigaya Y Matsubara E Ikeda M Park JM Yasutake K Kawarabayashi T Okamoto K Shoji M Abeta amyloidosis induces the initial stage of tau accumulation in APP(Sw) mice Neurosci Lett 2001 299 169 172 10.1016/S0304-3940(00)01767-5 11165762

[B31] Chin J Palop J Puoliväli J Massaro C Bien-Ly N Gerstein H Scearce-Levie K Masliah E Mucke L Fyn kinase induces synaptic and cognitive impairments in a transgenic mouse model of Alzheimer's disease J Neurosci 2005 25 9694 9703 10.1523/JNEUROSCI.2980-05.2005 16237174 PMC6725734

[B32] Ho GJ Hashimoto M Adame A Izu M Alford MF Thal LJ Hansen LA Masliah E Altered p59Fyn kinase expression accompanies disease progression in Alzheimer's disease: implications for its functional role Neurobiol Aging 2005 26 625 635 10.1016/j.neurobiolaging.2004.06.016 15708437

[B33] Lambert MP Barlow AK Chromy BA Edwards C Freed R Liosatos M Morgan TE Rozovsky I Trommer B Viola KL Diffusible, nonfibrillar ligands derived from Abeta1-42 are potent central nervous system neurotoxins Proc Natl Acad Sci USA 1998 95 6448 6453 10.1073/pnas.95.11.6448 9600986 PMC27787

[B34] Williamson R Usardi A Hanger DP Anderton BH Membrane-bound beta-amyloid oligomers are recruited into lipid rafts by a fyn-dependent mechanism FASEB J 2008 22 1552 1559 10.1096/fj.07-9766com 18096814

[B35] Lesort M Greendorfer A Stockmeier C Johnson GV Jope RS Glycogen synthase kinase-3beta, beta-catenin, and tau in postmortem bipolar brain J Neural Transm 1999 106 1217 1222 10.1007/s007020050235 10651115

[B36] Hata R Masumura M Akatsu H Li F Fujita H Nagai Y Yamamoto T Okada H Kosaka K Sakanaka M Sawada T Up-regulation of calcineurin Abeta mRNA in the Alzheimer's disease brain: assessment by cDNA microarray Biochem Biophys Res Commun 2001 284 310 316 10.1006/bbrc.2001.4968 11394878

[B37] Zhong W Yamagata H Taguchi K Akatsu H Kamino K Yamamoto T Kosaka K Takeda M Kondo I Miki T Lymphocyte-specific protein tyrosine kinase is a novel risk gene for Alzheimer disease J Neurol Sci 2005 238 53 57 10.1016/j.jns.2005.06.017 16109429

[B38] Omri B Blancher C Neron B Marty MC Rutin J Molina TJ Pessac B Crisanti P Retinal dysplasia in mice lacking p56lck Oncogene 1998 16 2351 2356 10.1038/sj.onc.1201761 9620552

[B39] Schweers O Schonbrunn-Hanebeck E Marx A Mandelkow E Structural studies of tau protein and Alzheimer paired helical filaments show no evidence for beta-structure J Biol Chem 1994 269 24290 24297 7929085

[B40] Lee G Newman ST Gard DL Band H Panchamoorthy G Tau interacts with src-family non-receptor tyrosine kinases J Cell Sci 1998 111 3167 3177 9763511 10.1242/jcs.111.21.3167

[B41] Bhaskar K Yen S Lee G Disease-related modifications in tau affect the interaction between Fyn and Tau J Biol Chem 2005 280 35119 35125 10.1074/jbc.M505895200 16115884

[B42] Reynolds CH Garwood CJ Wray S Price C Kellie S Perera T Zvelebil M Yang A Sheppard PW Varndell IM Phosphorylation regulates tau interactions with Src homology 3 domains of phosphatidylinositol 3-kinase, phospholipase Cgamma1, Grb2, and Src family kinases J Biol Chem 2008 283 18177 18186 10.1074/jbc.M709715200 18467332

[B43] Agarwal-Mawal A Qureshi HY Cafferty PW Yuan Z Han D Lin R Paudel HK 14-3-3 connects glycogen synthase kinase-3 beta to tau within a brain microtubule-associated tau phosphorylation complex J Biol Chem 2003 278 12722 12728 10.1074/jbc.M211491200 12551948

[B44] Theus MH Wei L Francis K Yu SP Critical roles of Src family tyrosine kinases in excitatory neuronal differentiation of cultured embryonic stem cells Exp Cell Res 2006 312 3096 3107 10.1016/j.yexcr.2006.06.022 16859680

[B45] Nelson PT Stefansson K Gulcher J Saper CB Molecular evolution of t protein: Implications for Alzheimer's disease J Neurochem 1996 67 1622 1632 10.1046/j.1471-4159.1996.67041622.x 8858947

[B46] Brandt R Léger J Lee G Interaction of tau with the neural plasma membrane mediated by tau's amino-terminal projection domain J Cell Biol 1995 131 1327 1340 10.1083/jcb.131.5.1327 8522593 PMC2120645

[B47] Sun W Qureshi HY Cafferty PW Sobue K Agarwal-Mawal A Neufield KD Paudel HK Glycogen synthase kinase-3beta is complexed with tau protein in brain microtubules J Biol Chem 2002 277 11933 11940 10.1074/jbc.M107182200 11812770

[B48] Alonso A Zaidi T Novak M Grundke-Iqbal I Iqbal K Hyperphosphorylation induces self-assembly of tau into tangles of paired helical filaments/straight filaments Proc Natl Acad Sci USA 2001 98 6923 6928 10.1073/pnas.121119298 11381127 PMC34454

[B49] Ittner LM Ke YD Delerue F Bi M Gladbach A van Eersel J Wolfing H Chieng BC Christie MJ Napier IA Dendritic function of tau mediates amyloid-beta toxicity in Alzheimer's disease mouse models Cell 2010 142 387 397 10.1016/j.cell.2010.06.036 20655099

[B50] Mulot SFC Hughes K Woodgett JR Anderton BH Hanger DP PHF-tau from Alzheimer's brain comprises four species on SDS-PAGE which can be mimicked by in vitro phosphorylation of human brain tau by glycogen synthase kinase-3b FEBS Lett 1994 349 359 364 10.1016/0014-5793(94)00702-0 8050597

[B51] Giannini A Bijlmakers MJ Regulation of the Src family kinase Lck by Hsp90 and ubiquitination Mol Cell Biol 2004 24 5667 5676 10.1128/MCB.24.13.5667-5676.2004 15199125 PMC480905

[B52] Brion JP Neurofibrillary tangles and Alzheimer's disease Eur Neurol 1998 40 130 140 10.1159/000007969 9748670

[B53] Boyle WJ van der Geer P Hunter T Phosphopeptide mapping and phosphoamino acid analysis by two-dimensional separation on thin-layer cellulose plates Methods Enzymol 1991 201 110 149 full_text 1943760 10.1016/0076-6879(91)01013-r

[B54] Laemmli UK Cleavage of structural proteins during the assembly of the head of bacteriophage T4 Nature 1970 227 680 685 10.1038/227680a0 5432063

[B55] Davis DR Brion JP Couck AM Gallo JM Hanger DP Ladhani K Lewis C Miller CCJ Rupniak T Smith C Anderton BH The phosphorylation state of the microtubule-associated protein tau as affected by glutamate, colchicine and beta-amyloid in primary rat cortical neuronal cultures Biochem J 1995 309 941 949 7639714 10.1042/bj3090941 PMC1135722

