# Supplementary material for: Tyrosine Phosphorylation of Tau by the Src Family Kinases Lck and Fyn
Source: Mol Neurodegener. 2011 Jan 26;6:12. doi: 10.1186/1750-1326-6-12 (PMC3037338; doi:10.1186/1750-1326-6-12)
Supplement: Additional file 1 — Table S1. "Tyrosine-phosphorylated peptides identified by LC-MS/MS in tryptic digests of 2N4R tau after phosphorylation by Lck and Fyn." [file 1750-1326-6-12-S1.PDF]

Table S1.

Tyrosine-phosphorylated peptides identified by LC-MS/MS in tryptic digests of 2N4R tau after phosphorylation by Lck and Fyn.

Residues	Sequence[a]	Site Identified	m/z (charge)	Lck[b]	Fyn[b]
6-23	QEFEVoxMEDHAGTpYGLGDR	18	1075.43 (2+)	+	+
6-23	QEFEVoxMEDHAGTpYGLGDR	18	717.28 (3+)	+	+
24-44	KDQGGpYpToxMHQDQEGDTDAGLK	29	797.32 (3+)	ss	ss
195-209	SGpYSSPGSPGTPGSR	197	737.28 (2+)	ss	ss
299-317	HVPGGGSVQIVpYKPVDLSK	310	1030.53 (2+)	+	ss
386-406	TDHGAEIVpYKSPVVSGDTSPR	394	765.69 (3+)	+	+

[a] pY, phosphorylated tyrosine; oxM, oxidised methionine

[b] +, phosphorylated residue identified by MS/MS; ss, survey scan (LC-MS/MS) or MALDI-ToF MS only
