# Supplementary material for: Tyrosine Phosphorylation of Tau by the Src Family Kinases Lck and Fyn
Source: Mol Neurodegener. 2011 Jan 26;6:12. doi: 10.1186/1750-1326-6-12 (PMC3037338; doi:10.1186/1750-1326-6-12)
Supplement: Additional file 2 — Figure S1. "LC-MS/MS spectrum of a phosphopeptide of Mr 2148.83 Da from Lck-phosphorylated tau." [file 1750-1326-6-12-S2.PDF]

Figure S1.

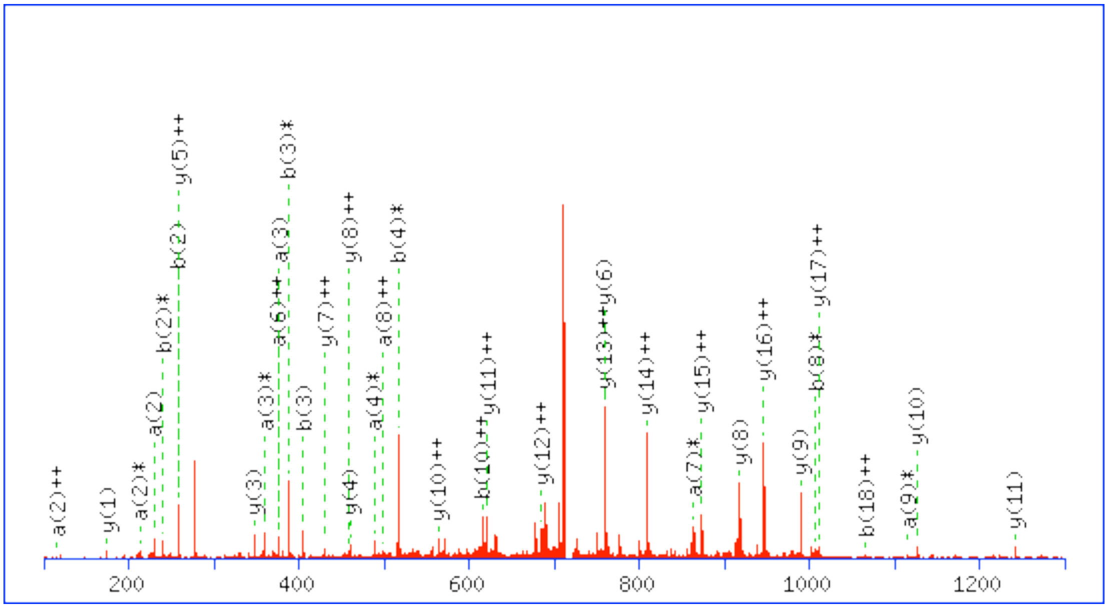

QEFEVMEDHAGTY*GLGDR

Fig. S1. **LC-MS/MS spectrum of a phosphopeptide of Mr 2148.83 Da from Lck-phosphorylated tau.** Y* denotes phosphorylated tyrosine, and the methionine (M) is oxidised. Analysis of this spectrum is shown in Table S2.
