# Supplementary material for: Tyrosine Phosphorylation of Tau by the Src Family Kinases Lck and Fyn
Source: Mol Neurodegener. 2011 Jan 26;6:12. doi: 10.1186/1750-1326-6-12 (PMC3037338; doi:10.1186/1750-1326-6-12)
Supplement: Additional file 3 — Table S2. "Analysis of LC-MS/MS spectrum shown in additional File 2: Figure S1." [file 1750-1326-6-12-S3.PDF]

Table S2.

Analysis of LC-MS/MS spectrum shown in additional File 2: Fig. S1. Ions identified are shown in italics, where at least one ion confirms the positions of 16 of the 18 residues including the phosphotyrosine. Identification of ions 5 and 6 in the y-series differ by the mass of phosphotyrosine (243), demonstrating that tyrosine 18 is phosphorylated. The sequence shows that the peptide was derived from residues 6-23 of tau, with phosphorylation of Tyr18 and oxidation of Met11.

#	a	b	b*	b^{++}	Seq.	y	y*	y^{++}	#
**1**	101.07	129.07	112.04	65.04	**Q**	2149.85	2132.83	1075.43	**18**
**2**	**230.11**	**258.11**	**241.08**	129.56	**E**	2021.80	2004.77	**1011.40**	**17**
**3**	**377.18**	**405.18**	**388.15**	203.09	**F**	1892.75	1875.73	**946.88**	**16**
**4**	506.23	534.22	**517.19**	267.61	**E**	1745.68	1728.66	**873.35**	**15**
**5**	605.29	633.29	**616.26**	317.15	**V**	1616.64	1599.62	**808.82**	**14**
**6**	752.33	780.32	763.30	390.67	**oxM**	**1517.57**	1500.55	**759.29**	**13**
**7**	881.37	909.37	892.34	455.19	**E**	1370.54	**1353.51**	**685.77**	**12**
**8**	996.40	1024.39	**1007.37**	512.70	**D**	**1241.50**	1224.47	**621.25**	**11**
**9**	1133.46	1161.45	1144.43	581.23	**H**	**1126.47**	1109.44	**563.74**	**10**
**10**	1204.49	1232.49	1215.46	**616.75**	**A**	**989.41**	972.38	495.21	**9**
**11**	1261.52	1289.51	1272.48	645.26	**G**	**918.37**	901.35	**459.69**	**8**
**12**	1362.56	1390.56	1373.53	695.78	**T**	861.35	844.32	**431.18**	**7**
**13**	1605.59	1633.59	1616.56	817.30	**pY**	**760.30**	743.28	380.66	**6**
**14**	1662.61	1690.61	1673.58	845.81	**G**	**517.27**	500.25	**259.14**	**5**
**15**	1775.70	1803.69	1786.67	902.35	**L**	**460.25**	443.23	230.63	**4**
**16**	1832.72	**1860.72**	1843.69	930.86	**G**	**347.17**	330.14	174.09	**3**
**17**	1947.75	1975.74	1958.72	988.37	**D**	290.15	273.12	145.58	**2**
