# Supplementary material for: Tyrosine Phosphorylation of Tau by the Src Family Kinases Lck and Fyn
Source: Mol Neurodegener. 2011 Jan 26;6:12. doi: 10.1186/1750-1326-6-12 (PMC3037338; doi:10.1186/1750-1326-6-12)
Supplement: Additional file 4 — Table S3. "Identification by mass spectrometry of tau phosphopeptides in spots from 2D phosphopeptide maps." [file 1750-1326-6-12-S4.PDF]

Table S3.

Identification by mass spectrometry of tau phosphopeptides in spots from 2D phosphopeptide maps.

Spot Number[a]	Method of Identification	Residues	Sequence[b]	m/z (charge)	Site identified
1	LC-MS/MS	6-23	QEFEVMEDHAGTpYGLGDR	1068.98 (2+)	18
2	LC-MS/MS	6-24	QEFEVMEDHAGTpYGLGDRK	754.35 (3+)	18
3	LC-MS/MS	6-23	QEFEVoxMEDHAGTpYGLGDR	717.28 (3+)	18
4	LC-MS/MS	6-24	QEFEVoxMEDHAGTpYGLGDRK	759.98 (3+)	18
5	LC-MS/MS	195-209	SGpYSSPGSPGTPGTPGSR	737.28 (2+)	197
1f	MALDI-ToF MS	6-23	QEFEVMEDHAGTpYGLGDR	711.1 (3+)	18
2f	MALDI-ToF MS	6-24	QEFEVMEDHAGTpYGLGDRK	1130.7 (2+)	18
3f	LC-MS/MS	6-23	QEFEVoxMEDHAGTpYGLGDR	1075.52 (2+)	18

[a] Spots 1-5: from Lck-phosphorylated tau (Fig. 3A); spots 1f-3f: from Fyn-phosphorylated tau (Fig. 3B)

[b] pY, phosphorylated tyrosine; oxM, oxidised methionine
