# Supplementary material for: Tyrosine Phosphorylation of Tau by the Src Family Kinases Lck and Fyn
Source: Mol Neurodegener. 2011 Jan 26;6:12. doi: 10.1186/1750-1326-6-12 (PMC3037338; doi:10.1186/1750-1326-6-12)
Supplement: Additional file 5 — Figure S2. "2D Phosphopeptide mapping of tau C- and N-terminal constructs phosphorylated by Lck." [file 1750-1326-6-12-S5.PDF]

Figure S2

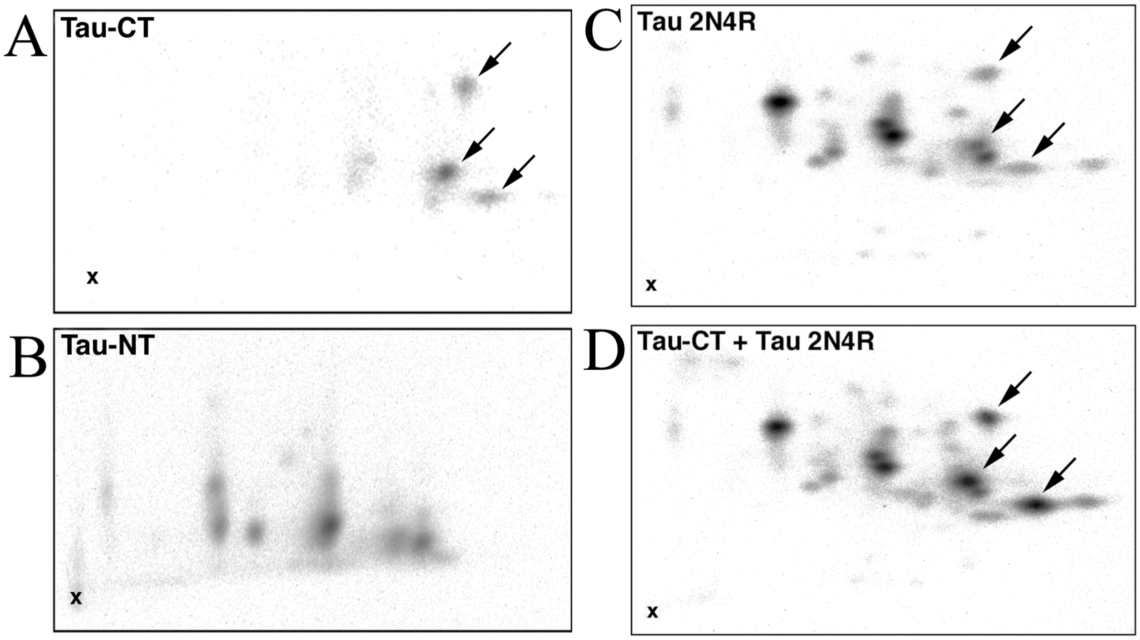

Fig. S2. **2D Phosphopeptide mapping of tau C- and N-terminal constructs phosphorylated by Lck.** *A,* Tau C-terminal construct (Tau-CT). *B,* Tau N-terminal construct (Tau-NT). *C,* 2N4R tau. *D,* 'Mixed' 2D phosphopeptide map of a sample of 2N4R tau mixed with Tau-CT construct. *Arrows* mark the three main peptide spots on the Tau-CT map, their proposed equivalents on the 2N4R tau map and confirmation of the equivalents that appear darker in the "Mixed" 2D map.
